# Line × tester analysis for yield and its components of some domestic okra (*Abelmoschus esculentus* L. Moench) lines

**DOI:** 10.1038/s41598-026-52940-7

**Published:** 2026-05-21

**Authors:** M. Y. Abed, A. M. El-Shoura

**Affiliations:** 1 Vegetables Research Department, Giza, Egypt; 2 Horticulture Research Institute, Agricultural Research Center, Giza, Egypt

**Keywords:** Okra, Line, Tester, Heterosis, Combining ability, Genetics, Plant sciences

## Abstract

The production of high-yielding and enhanced-quality cultivars of okra has attracted interest from the industry of vegetable seeds production. Thus, field experiments were conducted at El-Baramon Research Station, Horticulture Research Institute. Twenty F_1_ hybrids were generated by crossing ten female lines and two male testers using a line × tester mating design. Five hybrids, (L8×T2, L4×T2, L8×T1, L4×T1 and L5×T1), showed higher pod yield compared to the other hybrids. Most of the F_1_ hybrids revealed significant heterosis over mid or better parent for yield traits. The best positive heterotic cross was L8×T2 (24.27%) and (17.28%) over mid and better parent, respectively, for pod yield per plant in a desirable direction. Furthermore, the assessments of combining ability variances revealed the predominance of non-additive genetic variance over additive variance in the inheritance of these traits. Specific lines, e.g., L4, L5, L8 and L10 showed favorable *gca* effects and superior individual performances for pod yield per plant. Cross combinations such as L9×T1, L3×T2, L4×T2, L2×T2 and L5×T1 exhibited desirable *sca* effects for pod yield per plant along with superior individual performances. The results exhibited that the values of the non-additive genetic variance (*σ*^*2*^*D*) were greater than those of additive genetic variance (*σ*^*2*^*A*) for all the traits indicating the major role of non-additive gene effect in the inheritance of the studied traits. Furthermore, the narrow sense heritability varied from 0.49 to 47.37% for pod length and total yield, respectively. To sum up, this study identified superior inbreds, produced promising hybrids, showed desirable specific combining ability action and provided high estimates of useful heterosis, which could be utilized for okra hybrid production.

## Introduction

Okra (*Abelmoschus esculentus* (L.) Moench), which is native to tropical and subtropical Africa, is a major vegetable crop due to its nutritional value, ease of production, and export potential. Okra is grown all over the world, mostly for its edible immature and tender pods. It is consumed as a cooked vegetable which contains good amounts of calcium, potassium, and other minerals as well as other vitamins such as A, B and C^22^. Okra breeding efforts to generate high-yielding and better quality cultivars have been given affirmation by the industry of production of vegetable seeds. However, plants have shown low overall yields due to a lack of adaptive genotypes, a small genetic range of current varieties, and genetic erosion of some domestic cultivars^2^. The ultimate goal of most breeding programs is developing high yield hybrids, which depend on the identification of inbred lines with high general and specific combining ability. Therefore, okra breeders are searching for elite inbred lines with great general and specific combining ability effects to utilize as parents for new promising hybrids. Furthermore, exploitation of heterosis plays a major role for making general improvement of pod yield and its related traits as the first step in okra hybrid production^10^. The second step is to produce cross combinations using a suitable mating fashion, to exploit the amount of heterosis for various economic traits in okra and the inheritance pattern of recommended traits^7–9^.

Line × tester mating design is a valuable tool to calculate both general combining ability (GCA) for lines and testers ,  specific combining ability (SCA) of each hybrid and to estimate gene actions controlling the inheritance of quantitative traits. It provides data about the importance of dominance and additive components of genetic variances. Additionally, it is helpful in estimation of various types of gene effects that are major in the expression of qualitative and quantitative characters^14^. The specific combining ability, which represents the non-additive gene effect, is an important component used in heterosis breeding. Combining ability studies are more reliable as they provide useful information for the selection of parents in terms of the performance of the hybrids and elucidate the nature and magnitude of various types of gene actions involved in the expression of quantitative traits. Understanding these genetic attributes is crucial for identifying promising parental lines and developing high-yielding hybrids from domestic okra germplasm.

The performance of parental lines is directly proportional to their general combining ability GCA effects in parents, who show high general combining ability effects for various characters. The estimations of specific combining ability (SCA) effects of excellent crossings have specific combining ability effects for pod yield and associated traits. The specific combining ability is the response of the non-additive gene effect, which is an essential component in heterosis breeding. The heterosis of low × high combiners may be caused by dominant and additive interactions. The hybrids of both poor combiners show high SCA for yield plant^− 1^, however, both parental lines show negative significant GCA^29,46^. The non-additive gene effect is integrated in the genetic architecture of different characteristics utilized in okra. The combining abilities are analyzed utilizing a line x tester mating design. Analyses of variance for *GCA* and *SCA* for all characters are executed and the mean squares due to GCA and *SCA* indicate the variability among GCA and SCA for all studied traits. The variance due to lines would be significant for some characters which differs from the variance due to testers.  Additionally, the variances due to hybrids could be significant for few or all traits. The significant variance explains the existence of good amount of genetic variation among parents and their hybrids for their respective traits. The selection of breeding programs for yield and its components improvement is possible upon the nature of gene actions influencing quantitative characters.

The assessment of the combining ability in okra by the line x tester scheme revealed the best combiner of parents and distinguished transgressive segregants from succeeding generations of segregating individuals^36,41^. Thus, it is crucial for the plant breeders in choice of suitable parents (GCA effects) for hybridization program. Moreover, the heterosis studies of economical traits have been investigated by this mating design and provided valuable information regarding cross combinations (SCA effects) to be exploited commercially^9,29,45^. Additionally, it is an efficient method to reveal the gene action of the yield and yield attributing traits in okra breeding^30,43^.

The genetic variance among the studied cultivars is controlled by gene expression, which affects plant growth, architecture, flowering, pod formation and development. Furthermore, auxins modulate various signaling pathways by regulating gene expression and interacting with other phytohormones, thereby influencing physiological processes that generate the genetic variances and plant responses to environmental conditions^3^.

The main objectives of this study were to estimate the general (GCA) and specific (SCA) combining ability effects, genetic parameters, heterosis, and type of gene action involved in the expression of yield and its contributing traits and to identify superior inbred lines to be used in okra breeding programs and to determine the superior hybrids for productivity and pod traits.

## Materials and methods

This study was carried out during 2022 and 2023 at El-Baramon Research Station, Horticulture Research Institute, Egypt. In this experiment, the parental cultivars are comprised of 12 parents of okra (10 lines and 2 testers). They were developed from a previous breeding program in Horticulture Research Institute, Agricultural Research Center, Egypt.

Seeds of okra cultivars were planted on 14^th^ March 2022 and 2023 in both years of the study. Three seeds per hill were dibbled with a spacing of 30 cm between plants, and thinning was done to leave one stable and robust seedling per hill. The evaluation experiment was laid out in a randomized complete block design (RCBD) with three replications to assess the performance of 12 parents of okra, which involved 10 lines, 2 testers and 20 cross combinations were developed by crossing ten lines and two testers in line × tester mating design. The gab between replications was 1 m, while each plot consisted of 4 rows 0.6 cm wide × 5 m long,  and a plant-to plant spacing of 30 cm was adopted. Data on nine quantitative traits in okra viz. plant height (P.H., cm), number of branches plant^− 1^ (N.B.P.), days to flowering (D.F., days), pod length (P.L., cm), pod diameter (P.D., cm), average pod weight (A.P.W., g), number of pods plant^− 1^ (N.P.P), pod yield plant^− 1^ (P.Y.P, g), and total yield feddan^− 1^ (T.Y.F., ton) were recorded foren randomly selected plants in each plot. To obtain the successful crop of okra, standard agricultural practices were followed according to commercial recommendations.

The significance of differences among genotypes for all studied traits was estimated by conducting an analysis of variance (ANOVA) using the procedure proposed by Snedecor and Cochran^42^. Least significant of differences (LSD), at 1% and 5% of probability, was implemented for mean comparisons.

The line × tester analysis of the genotypes and partition of mean squares were conducted as outlined by Kempthorne^19^.

The performance of the cross combinations was estimated based on the heterosis versus mid parent and better parents, following the method calculated by Fehr^12^.

$${\mathrm{Heterosis}}\;{\mathrm{versus}}\;{\mathrm{mid}}\;{\mathrm{parent}}\,\left( {{\mathrm{M}}.{\mathrm{P}}.{\mathrm{H}}} \right){\text{ }} = {\text{ }}\left( {{\mathrm{F}}_{{\mathrm{1}}} - {\mathrm{M}}.{\mathrm{P}}/{\mathrm{M}}.{\mathrm{P}}} \right) \times {\mathrm{1}}00.$$

$${\mathrm{Heterosis}}\;{\mathrm{versus}}\;{\mathrm{better}}\;{\mathrm{parent}}\;\left( {{\mathrm{B}}.{\mathrm{P}}.{\mathrm{H}}} \right)\, = \,\left( {{\mathrm{F}}_{{\mathrm{1}}} - {\mathrm{B}}.{\mathrm{P}}/{\mathrm{B}}.{\mathrm{P}}} \right) \times {\mathrm{1}}00.$$

General combining ability (GCA) effects and specific combining ability (SCA) effects of cross combination were calculated following the methodology suggested by Singh and Chaudhary^40^.

The linear models assumed are:

$${\mathrm{Yijk}} = {\text{ }}\mu + {\mathrm{gi}} + {\mathrm{gj}} + {\mathrm{Sij}} + {\mathrm{eijk}}$$

Where,

Yijk = value of the ij^th^ observations of the cross involving i^th^ line and j^th^ tester in k^th^ replication;

µ = general mean (an effect common to all crosses in all the replications);

g_i_ = general combining ability (GCA) effect of i^th^ line;

g_j_ = general combining ability (GCA) effect of j^th^ tester;

s_ij_ = specifi c combining ability (SCA) effect of the cross involving ith line and jth tester;

e _ijk_ = error associated with ijth observation;

i = i_th_ line (1, 2, … 10);

j = j^th^ tester (1, 2,); and k = k^th^ replication (1, 2, 3).

**Estimation of** ***GCA*** **effects**:

*gca* (Lines): g_i_ = $\:\frac{\mathrm{x}\mathrm{i}..}{\mathrm{l}\mathrm{r}}$ - $\:\frac{\mathrm{x}..}{\mathrm{l}\mathrm{t}\mathrm{r}}$, *gca* (Tester): gi = $\:\frac{\mathrm{x}.\mathrm{i}.}{\mathrm{l}\mathrm{r}}$ - $\:\frac{\mathrm{x}\dots\:}{\mathrm{l}\mathrm{t}\mathrm{r}}$ .

**Estimation of** ***SCA*** **effects**:

$$S_{{ij}} = \frac{{{\mathrm{xij}}{\mathrm{.}}}}{{\mathrm{r}}} - \frac{{{\mathrm{xi}}..}}{{{\mathrm{tr}}}} - \frac{{{\mathrm{x}}.{\mathrm{i}}.}}{{{\mathrm{lr}}}} + \frac{{{\mathrm{x}} \ldots }}{{{\mathrm{ltr}}}}$$

Estimations of additive (σ^2^A) and non-additive, including dominance, (σ^2^D) components of variance were computed following formulae by Singh and Chaudhary^40^. Since okra is a self-pollinated crop and in the formulae as equal to 1.

$$\sigma ^{{\mathrm{2}}} {\text{gca }} = {\text{ }}\left[ {\left( {{\mathrm{1}}\, + \,{\mathrm{F}}} \right)/{\mathrm{4}}} \right]{\text{2 }}\sigma ^{{\mathrm{2}}} {\mathrm{A}},$$

$$\sigma ^{{\mathrm{2}}} {\text{sca }} = {\text{ }}\left[ {\left( {{\mathrm{1}}\, + \,{\mathrm{F}}} \right)/{\mathrm{4}}} \right]{\text{2 }}\sigma ^{{\mathrm{2}}} {\text{D }}.$$

Narrow (*h*^*2*^_*n.s*_ %) and broad sense (*h*^*2*^_*b.s*_*%*) heritabilities were recorded accordance to the following equations as per Mather and Jinks^23^:

$$h^{2} _{{n.s}} \% {\text{ }} = {\text{ }}\{ \sigma ^{{\mathrm{2}}} {\text{A }}/{\text{ }}\sigma ^{{\mathrm{2}}} {\mathrm{A}}\, + \,\sigma ^{{\mathrm{2}}} {\mathrm{D}}\, + \,\sigma ^{{\mathrm{2}}} {\mathrm{E}}\} {\text{ x 1}}00,$$

$$h^{2} _{{b.s}} \% = {\text{ }}\{ \sigma ^{{\mathrm{2}}} {\mathrm{A}}\, + \,\sigma ^{{\mathrm{2}}} {\text{D }}/{\text{ }}\sigma ^{{\mathrm{2}}} {\mathrm{A}}\, + \,\sigma ^{{\mathrm{2}}} {\mathrm{D}}\, + \,\sigma ^{{\mathrm{2}}} {\mathrm{E}}\} {\text{ x 1}}00.$$

The average degree of dominance was calculated by the formula presented by Mather and Jinks^23^:

$${\mathrm{D}}.{\mathrm{d}}.{\text{ }} = {\text{ }}\left( {{\mathrm{H}}/{\mathrm{D}}} \right)^{{{\mathrm{1}}/{\mathrm{2}}}} .$$

where:

H = Components of variation due to dominance.

D = Components of variation due to additive effects.

Contribution of Line (L), Testers (T) and their interactions to total variance: Proportional contribution of lines, testers and their interactions (L × T) were calculated using the formulae by^40^:

$${\mathrm{Contribution}}\;{\mathrm{of}}\;{\mathrm{line}}\, = \,{\text{SS }}\left( {\mathrm{L}} \right){\text{ }}/{\text{ SS }}\left( {{\mathrm{Crosses}}} \right){\text{ }} \times {\text{ 1}}00.$$

$${\mathrm{Contribution}}\;{\mathrm{of}}\,{\mathrm{tester}}\, = \,{\text{SS }}\left( {\mathrm{T}} \right){\text{ }}/{\text{ SS }}\left( {{\mathrm{Crosses}}} \right){\text{ }} \times {\text{ 1}}00.$$

$${\mathrm{Contribution}}\;{\mathrm{of}}\;{\text{line }} \times {\text{ tester}}\, = \,{\text{SS }}\left( {{\text{L }} \times {\text{ T}}} \right){\text{ }}/{\text{ SS }}\left( {{\mathrm{Crosses}}} \right){\text{ }} \times {\mathrm{1}}00.$$

## Results and discussion

A detailed understanding of the inheritance of the important traits in okra is essential for to the improving of the yield and pod characters. Identifying exemplary parents and developing and promotion a hybrid will not only increase the yield, but also serve as sources of genetic diversity useful for continuous improvement programs.

The obtained results in Table 1 revealed that the mean squares due to parents and their crosses for various traits showed significant differences between parents as well as between crosses for most traits which suggested the existence of considerable amount of genetic diversity in the parental lines. Emphasized the differential performance between the parents and their crosses which is an indicator of substantial significant genetic variation that could be explored for future genetic improvement^14^. Such findings indicate that hybrid combinations can outperform their parental lines and contribute significantly to yield improvement.

Furthermore, the analysis of variance for the studied traits in Table 1 demonstrated significant differences between crosses for all measured traits except for number of branches plant^−1^and pod diameter, which indicates the existence of variation between the produced crosses. Moreover, the results displayed that mean squares due to lines and testers were significant for majority of the traits, indicating significant variability between lines and testers. Notably, mean squares due to lines were highly significant for most studied traits, demonstrating that lines played a great role in determining trait expression. Tester effects were also significant for several traits, emphasizing the importance of selecting appropriate testers in okra breeding programs. There was significant interference in the mean squares for nine traits (plant height, number of branches plant^− 1^, days to flowering, pod length, pod diameter, average pod weight, number of pods plant^− 1^, pod yield plant^−1^and total yield feddan^− 1^) and significant interference in the mean squares for three measured traits (pod length, pod diameter and total yield). The overlap among lines and testers indicates the need for continuous analysis of general and specific combining ability to evaluate parents that have successfully merged with each other. These results align with those of Satish et al.,^35^, and Yadav et al.,^50^. In our experiment, line × tester effects were highly significant for all traits except for pod length and pod diameter, which means that breeding strategies can be used of both additive and non-additive (dominance) gene actions. In general, the combined interpretation of the results and discussion indicatethat the used okra germplasm possesses sufficient genetic variability and combining ability. The selection of superior parents and hybrids based on their performance and combining ability could lead to substantial improvements in yield and related traits in future okra breeding programs.

**Table 1:** Line x tester analysis and mean squares of all genotypes for the studied traits of okra.

S. V	d.f	*P*.H. (cm)	*N*.B.*P*.	D.F. (days)	*P*.L. (cm)	*P*.D (cm)	A.*P*.W. (g)	*N*.*P*.*P*.	*P*.Y.*P*.	T.Y.F. (ton)
Replications	2	20.40^NS^	0.04^NS^	2.91^NS^	0.01^NS^	0.01^NS^	0.02^NS^	31.39^NS^	46.79^NS^	0.01^NS^
Genotypes	31	1058.90^**^	2093^**^	84.32^**^	4.64^**^	0.09^**^	2.62^**^	224.33^**^	8846.45^**^	1.74^**^
Parents (P.)	11	1584.17^**^	2.21^**^	87.06^**^	4.46^**^	0.07^**^	2.48^**^	214.11^**^	5828.59^**^	1.07^**^
Crosses (C.)	19	799.08^**^	2.25 ^NS^	66.43^**^	4.78^**^	0.10	2.81^**^	196.38^**^	10286.19^**^	2.18^**^
P. vs. C.	1	217.46 ^NS^	23.77 ^NS^	395.67 ^NS^	1.08 ^NS^	0.003 ^NS^	0.50 ^NS^	867.76 ^NS^	12101.70 ^NS^	2.66 ^NS^
Lines	9	1011.58^**^	2.78	81.63^**^	9.88^**^	0.15^*^	3.42^**^	330.34^**^	20176.81^**^	4.33^**^
Tester	1	3781.51^**^	1.18 ^NS^	84.78^**^	0.68 ^NS^	0.02 ^NS^	5.83^**^	150.75^**^	881.67^**^	0.06 ^NS^
Lines x Tester	9	255.19^**^	2.05^**^	49.20^**^	0.14 ^NS^	0.07 ^NS^	1.85^**^	67.50^**^	1440.52^**^	0.27^**^
Error	62	34.74	0.42	25.89	0.04	0.01	0.06	23.48	55.30	0.01

P.H.= plant height (cm); N.B.P.= number of branches plant^− 1^; D.F.= days to flowering (days); P.L.= pod length (cm); P.D.: pod diameter (cm); A.P.W= average pod weight (g); N.P.P= number of pods plant^− 1^; P.Y.P.= pod yield plant^− 1^ and T.Y.F.= total yield feddan^− 1^ (ton), NS: non-significant, *, **: significant at 0.05 and 0.01 levels of probability, respectively.

### Mean performance

The mean performance of lines, testers and their cross combinations, revealed significant differences of mean performance between the various genotypes (lines, testers and F_1_ hybrids) for all measured characters under the study (Table 2). Such wide variability reflects the diverse genetic backgrounds of the parental lines and indicates substantial potential for genetic improvement through hybridization. Notably, plant height (P.H.), ranged from 133.77 cm (L5) to 2.04 cm (L3) between parents; and from 151.66 cm (L1xT2) to 206.55 cm (L3×T1) between crosses. Concerning to number of branches, the measurements were varied from 1.33 (L8) to 4.00 (L2 and L7). For cross combinations, it was observed that hybrids varied from 2.67 (L7 × T1), (L8 × T1), (L5 × T2) to 5.67 branches in (L7 × T2). Similar findings were mentioned by Tiwari et al.,^48^ and Srikanth et al.,^47^. For days to flowering (D.F.), The earliest parent in flowering was L5 (59.44 days) while the earliest flowering were shown by crosses L8×T2, L5×T2 and L5×T1 (56.00, 56.44 and 56.66 days), respectively. Rynjah et al.,^34^and Verma and Sood^49^) reported similar results. There was a wide variation between flowering times among the studied genotypes. Kharat et al.,^20^ and Mundhe et al.,^24^ reported similar wide variation among crosses for number of days to flowering in okra. The range of variation for pod length (P.L.) varied from 3.37 cm (T2) to 7.37 cm (L8). For F_1_ hybrids, pod length at maturity was noticed that range from 3.33 cm in cross (L3 × T2) to 7.60 cm in cross (L8 × T2). Abinaya et al.,^1^ and Chavan et al.,^6^ reported similar results for pod length. In respect to pod diameter (P.D.) it was noticed that parents ranged from 1.43 cm (L5) to 1.87 cm (L2, L8 and L10). For F_1_ hybrids, the pod diameter at maturity was noticed that varied from 1.43 cm in crosses (L3 × T1) and (L6 × T1) to 2.13 cm in cross (L10 × T2). Srikanth et al.,^47^ reported similar findings for pod diameter. Regarding yield attributed traits, viz. average pod weight; number of pods and total yield plant^− 1^, average pod weight (A.P.W.) was noticed to range from 4.23 (L9) to 7.27 g (L8). For hybrids, average pod weight varied from 3.53 in cross (L6 × T1) to 7.07 g in (L2 × T1). Sood et al.,^45^ and Rajneesh et al.,^33^ showed similar results for the aforementioned trait. For number of pods plant^− 1^(N.P.P.), it was observed to be minimum 42.11 (L9) and maximum 70.77 (L4). For F_1_ hybrids, it was noticed that hybrids ranged from 47.00 in (L9×T1) to 76.55 pods in (L2×T1). Similar results were reported by^5,7^. Also, pod yield plant^− 1^ (P.Y.P.) was observed that parents range from 235.30 (L6) to 383.40 (L4). For F_1_ hybrids, it was observed that hybrids varied from 215.67 in (L9×T2) to 441.33 in (L8×T2). Finally, for total yield feddan^− 1^ was noticed to range from 3.29 (L6) to 5.37 ton (L4). The extent of variation for total yield feddan^− 1^ was from 3.04 to 6.35 ton in crosses L9×T2 and L8×T1, respectively. Similar significant variation was found by^28,31,37^.

Generally, several hybrids, notably L4×T1, L8×T1, L4×T2, and L8×T2, consistently surpassed their respective parents for pod yield plant^− 1^ and total yield feddan^− 1^. This superiority suggests the effective exploitation of heterosis and confirms that yield in okra is a complex trait influenced by the cumulative performance of its components. Furthermore, significant line × tester interactions were detected for most traits, especially yield and its components, indicating the importance of specific combining ability. This reflects the contribution of non-additive gene action in the inheritance of these traits. The prominence of non-additive effects suggests that hybrid breeding strategies would be more effective than simple selection for improving yield performance in okra. In general, the combined interpretation of results and discussion indicates that the estimated okra germplasm possesses sufficient genetic variability and combining ability. The selection of superior parents and hybrids on the basis of their performance and combining ability could lead to substantial improvements in yield and related traits in future okra breeding programs.

**Table 2:** Mean performance of parents and their F_1_ hybrids for nine measured traits in okra.

Traits Geno.	*P*.H. (cm)	*N*.B.*P*.	D.F. (days)	*P*.L. (cm)	*P*.D. (cm)	A.*P*.W. (g)	*N*.*P*.*P*.	*P*.Y.*P*.	T.Y.F. (ton)
**Lines**									
L 1	153.33	3.67	68.88	4.67	1.67	5.13	55.44	296.80	4.16
L2	186.44	4.00	71.11	4.13	1.87	4.83	66.22	294.40	4.12
L3	204.11	3.33	65.55	3.63	1.57	4.40	47.33	264.60	3.70
L4	148.77	2.67	65.77	3.97	1.80	5.47	70.77	383.40	5.37
L5	133.77	1.67	59.44	6.37	1.43	6.63	51.33	342.10	4.79
L6	192.11	2.67	74.11	3.63	1.53	5.20	47.22	235.30	3.29
L7	179.33	4.00	63.55	4.37	1.70	4.53	60.44	292.80	4.10
L8	192.77	1.33	60.00	7.37	1.87	7.27	54.33	376.30	5.27
L9	140.77	3.33	67.99	3.6	1.60	4.23	42.11	289.20	4.26
L10	180.4	3.67	71.11	4.33	1.87	5.83	56.22	336.40	4.71
**Tester**									
T1	183.22	2.67	76.99	3.70	1.60	6.00	52.89	318.00	4.51
T2	159.11	2.61	63.77	3.37	1.80	5.47	64.00	334.00	4.54
**Crosses**									
L1×T1	179.66	5.33	68.22	4.53	1.80	6.33	69.11	340.33	4.77
L2×T1	192.66	3.33	70.55	4.23	1.80	6.47	76.55	310.33	4.37
L3×T1	206.55	4.33	66.33	3.70	1.43	4.10	51.11	259.33	3.63
L4×T1	168.66	4.67	58.99	3.70	1.57	3.93	72.66	403.33	5.64
L5×T1	156.88	3.67	56.66	5.70	1.63	5.57	57.22	366.33	5.08
L6×T1	192.88	5.00	63.66	3.40	1.43	3.53	48.44	313.00	4.38
L7×T1	191.77	2.67	62.89	4.23	1.70	4.77	63.66	296.33	4.16
L8×T1	158.00	2.67	69.44	7.63	2.00	6.63	56.11	418.00	6.35
L9×T1	176.22	3.67	58.11	4.10	1.73	4.60	47.00	266.67	3.74
L10×T1	199.00	4.67	68.66	4.17	1.77	6.60	60.77	356.00	5.01
L1×T2	151.66	4.00	64.22	4.77	1.60	5.40	60.55	331.33	4.65
L2×T2	166.44	3.33	61.77	4.47	1.57	5.70	70.22	341.67	4.79
L3×T2	179.55	4.67	63.44	3.33	1.73	5.60	62.66	313.67	4.40
L4×T2	154.55	3.67	57.88	4.13	1.57	5.37	71.33	439.00	6.09
L5×T2	165.22	2.67	56.44	6.47	1.50	6.27	65.11	350.67	4.91
L6×T2	165.11	4.00	60.77	3.67	1.63	6.17	55.22	334.33	4.68
L7×T2	181.11	5.67	64.55	4.47	1.63	5.33	66.55	291.00	4.12
L8×T2	161.44	4.33	56.00	7.60	1.93	7.07	60.78	441.33	6.17
L9×T2	155.66	4.33	65.66	4.47	1.90	5.67	56.55	215.67	3.04
L10×T2	182.77	3.33	69.00	4.07	2.13	6.20	65.99	347.67	4.91
SE±	0.965	0.117	0.719	0.001	0.0003	0.001	0.652	1.536	0.001
LSD 1%	12.78	1.40	11.03	0.40	0.18	0.52	10.51	16.13	0.22
LSD 5%	9.62	1.05	8.30	0.30	0.14	0.39	7.90	12.13	0.17

P.H.= plant height (cm); N.B.P.= number of branches plant^− 1^; D. F.= days to flowering (days); P.L.= pod length (cm); P.D.: pod diameter (cm); A.P.W.= average pod weight (g); N.P.P.= number of pods plant^− 1^; P.Y.P.= pod yield plant^− 1^ and T.Y.F.= total yield feddan^− 1^ (ton).

## Heterosis percentage

Heterosis was calculated as the percentage of decreases or increases of F_1_ hybrids magnitudes versus mid parents or better parent. In the present study the nature and values of heterosis for nine measured traits in okra in all 20 crosses are shown (Table 3). With respect to plant height, the hybrids with positive heterosis were considered as desirable for determining plant height. The magnitudes of heterosis ranged from – 15.96 (H8) to 18.78% (H9) and − 18.04 (H8) to 16.25% (H18) over mid parents and better parent, respectively. Crosses L9×T1 followed by L5×T2, were found to be the tallest for plant height versus mid parents in order to merit F_1_, whereas hybrids L8×T2 followed by L5×T1 were the tallest for plant height over better parent. Heterosis magnitude for aforementioned trait was reported by Patel^27^. In case of, number of branches plant^−1^L6×T1 and L4×T1 hybrids exhibited the greatest magnitudes of significant positive heterosis over mid parents and better parent, respectively. The heterosis magnitudes were ranged from − 0.30 to 85.24% and from − 9.26 to 87.27% over mid parents and better parent, respectively. The heterosis of this trait were reported by^32^. As for days to flowering, cross combinations with negative heterosis were considered as desirable estimations as they indicate earlier flowering habit^4,27^. In this respect, L9×T1 (− 19.84%) and L5×T1 (− 26.41) were recorded as negatively heterotic crosses for days to flowering over mid and better parent respectively, that are important for exploitation the earliness traits in okra. As for pod length, heterosis values for pod length ranged from − 7.36 (H6) to 43.34% (H18) and − 10.52 (H5) to 24.17% (H19) both over mid parents and better parent, respectively. Most of crosses exhibited significant positive heterosis versus mid parents and some of which for better parent, indicating a desirable heterosis for the crosses. Similar results reported by^25,29^. In respect of pod diameter, heterosis value over mid parents and better parent for this trait ranged from − 14.67 (H12) to 15.76% (H20) and from − 16.67 to 13.90% (H20), respectively. Cross combinations exhibited a little response versus mid and better parent. Identical obtained results for the pod diameter trait have been found by Solankey et al.,^44^ and Kumar et al.,^21^.

**Table 3:** Heterosis versus the mid parents and better parents for nine measured traits in okra.

Cross no.	Crosses	*P*.H. (cm)		*N*.B.*P*.		D.F. (days)		*P*.L. (cm)		*P*.D. (cm)	
		M.*P*	B.*P*	M.*P*	B.*P*	M.*P*	B.*P*	M.*P*	B.*P*	M.*P*	B.*P*
H1	L1 × T1	6.76 ^NS^	− 1.94 ^NS^	68.14^**^	45.33^**^	− 6.47 ^NS^	− 11.39^**^	8.11^**^	− 3.00^**^	9.76^**^	7.78^**^
H2	L2 × T1	4.24 ^NS^	3.34 ^NS^	− 0.30 ^NS^	− 16.75^**^	− 4.73 ^NS^	− 8.36^*^	7.91^**^	2.42^**^	3.45^**^	− 3.74^**^
H3	L3 × T1	6.65 ^NS^	1.20 ^NS^	44.33^**^	30.03^**^	− 6.93 ^NS^	− 13.85^**^	0.82^**^	0.00	− 10.06^**^	− 10.63^**^
H4	L4 × T1	1.60 ^NS^	− 7.95 ^NS^	71.91^**^	74.91^**^	− 17.36^**^	− 23.38^**^	− 3.65^**^	− 6.80^**^	− 7.65^**^	− 12.78^**^
H5	L5 × T1	− 0.01 ^NS^	14.38^**^	69.12^**^	37.45^**^	− 16.95^**^	− 26.41^**^	13.10^**^	− 10.52^**^	7.24^**^	1.88^**^
H6	L6 × T1	2.78 ^NS^	0.001 ^NS^	85.24^**^	87.27^**^	− 15.74^**^	− 17.31^**^	− 7.36^**^	− 8.11^**^	− 8.92^**^	− 10.63^**^
H7	L7 × T1	5.79 ^NS^	4.67 ^NS^	25.09^**^	− 33.25^**^	− 10.50^**^	− 18.31^**^	4.70^**^	− 3.20^**^	3.03^**^	0.00
H8	L8 × T1	− 15.96^**^	− 18.04^**^	33.5^**^	0.00	1.37 ^NS^	− 9.81^**^	37.73^**^	3.53^**^	14.94^**^	6.95^**^
H9	L9 × T1	18.78^**^	− 3.82 ^NS^	22.33^**^	10.21^**^	− 19.84^**^	− 24.52^**^	12.33^**^	10.81^**^	8.13^**^	8.13^**^
H10	L10 × T1	9.45^*^	8.61 ^NS^	47.32^**^	27.25^**^	− 7.28^*^	− 10.82^**^	3.73^**^	− 3.70^**^	1.72^**^	− 5.35^**^
H11	L1 × T2	− 2.92 ^NS^	− 4.68 ^NS^	27.39^**^	8.99^**^	− 3.18 ^NS^	− 6.77 ^NS^	18.66^**^	2.14^**^	− 8.05^**^	− 11.11^**^
H12	L2 × T2	− 3.67 ^NS^	− 10.73^*^	0.60	− 16.75^**^	− 8.41^*^	− 13.13^**^	19.20^**^	8.23^**^	− 14.67^**^	− 16.04^**^
H13	L3 × T2	− 1.13 ^NS^	− 12.03^*^	23.57^**^	10.21**	− 1.89 ^NS^	− 3.22 ^NS^	− 4.86^**^	− 8.26^**^	2.37^**^	− 3.89^**^
H14	L4 × T2	0.40 ^NS^	− 2.87 ^NS^	69.10^**^	37.45^**^	− 10.64^**^	− 10.48^*^	12.53^**^	4.03^**^	− 12.78^**^	− 12.78^**^
H15	L5 × T2	12.82^**^	3.84 ^NS^	24.77^**^	2.30^**^	− 8.39^*^	− 11.49^**^	32.85^**^	1.57^**^	− 7.14^**^	− 16.67^**^
H16	L6 × T2	− 5.98 ^NS^	− 14.05^**^	51.52^**^	49.81^**^	− 11.85^**^	− 18.00^**^	4.86^**^	1.10^**^	− 2.40^**^	− 9.45^**^
H17	L7 × T2	7.03 ^NS^	0.99 ^NS^	71.30^**^	41.75^**^	1.40 ^NS^	1.22 ^NS^	15.50^**^	2.29^**^	− 6.86^**^	− 9.45^**^
H18	L8 × T2	− 8.24 ^NS^	16.25^**^	19.80^**^	65.90^**^	− 9.52^*^	− 12.18^**^	43.34^**^	4.48^**^	4.89^**^	3.21^**^
H19	L9 × T2	3.81 ^NS^	− 2.17	45.79^**^	30.03^**^	− 1.66 ^NS^	− 3.43 ^NS^	28.00^**^	24.17^**^	11.76^**^	5.56^**^
H20	L10 × T2	7.66^*^	1.31 ^NS^	6.05^**^	− 9.26^**^	2.31 ^NS^	− 2.97 ^NS^	5.71^**^	− 6.01^**^	15.76^**^	13.90^**^
Minimum		− 15.96	− 18.04	− 0.30	− 9.26	− 10.84	− 26.41	− 7.36	− 10.52	− 14.17	− 16.67
Maximum		18.78	16.25	85.24	87.27	2.31	1.22	43.34	24.17	15.76	13.90

Cross no.	Crosses	A.*P*.W. (g)		*N*. *P*.*P*.		*P*.Y.*P*.		T.Y.F. (ton)	
		M.*P*	B.*P*	M.*P*	B.*P*	M.*P*	B.*P*	M.*P*	B.*P*
H1	L1 × T1	13.64^**^	5.50^**^	16.80^**^	24.66^**^	10.71^*^	7.02 ^NS^	42.81^**^	5.77^**^
H2	L2 × T1	19.37^**^	7.83^**^	28.53^**^	15.60^**^	1.35 ^NS^	− 2.41 ^NS^	1.16^**^	− 3.10^**^
H3	L3 × T1	− 21.15^**^	− 31.67^**^	2.00^**^	− 3.37 ^NS^	− 10.97^*^	− 18.45^**^	− 11.68^**^	− 19.51^**^
H4	L4 × T1	− 31.53^**^	34.50^**^	17.52^**^	2.67 ^NS^	15.01^**^	5.20 ^NS^	14.17^**^	5.03^**^
H5	L5 × T1	− 11.87^**^	− 15.99^**^	9.81^**^	8.19^*^	11.00^*^	7.08 ^NS^	9.25^**^	6.05^**^
H6	L6 × T1	− 36.96^**^	− 41.17^**^	− 3.24 ^NS^	− 8.41^*^	− 16.90^**^	− 6.65 ^NS^	12.31^**^	− 2.88^**^
H7	L7 × T1	35.90^**^	− 20.50^**^	12.33^**^	5.33 ^NS^	− 3.95 ^NS^	− 6.81 ^NS^	− 3.48^**^	− 7.76^**^
H8	L8 × T1	− 0.15 ^NS^	− 8.80^**^	4.66 ^NS^	3.28 ^NS^	20.41^**^	11.08 ^NS^	29.86^**^	20.49^**^
H9	L9 × T1	− 10.16^**^	− 23.33^**^	− 1.05 ^NS^	− 11.14^**^	− 12.16^*^	− 16.14^*^	− 14.81^**^	− 17.07^**^
H10	L10 × T1	11.49^**^	10.00^**^	11.38^**^	8.09^*^	8.80 ^NS^	5.83 ^NS^	8.68^**^	6.37^**^
H11	L1 × T2	1.89^**^	− 1.28^**^	1.39 ^NS^	− 5.39 ^NS^	5.05 ^NS^	− 0.80 ^NS^	6.90^**^	2.42^**^
H12	L2 × T2	10.68^**^	4.20^**^	7.85^*^	6.04 ^NS^	8.74 ^NS^	2.30 ^NS^	10.62^**^	5.51^**^
H13	L3 × T2	13.36^**^	2.38^**^	12.56^**^	− 2.09 ^NS^	4.80 ^NS^	− 6.09 ^NS^	6.80^**^	− 3.08^**^
H14	L4 × T2	− 1.83^**^	− 1.83^**^	5.85 ^NS^	0.79 ^NS^	22.39^**^	14.62^*^	22.78^**^	13.41^**^
H15	L5 × T2	− 12.89^**^	− 5.43^**^	12.90^**^	1.73 ^NS^	3.73 ^NS^	2.54 ^NS^	5.14^**^	2.51^**^
H16	L6 × T2	15.54^**^	18.65^**^	− 0.70 ^NS^	− 13.72^**^	17.39^**^	0.001 ^NS^	19.39^**^	3.08^**^
H17	L7 × T2	6.60^**^	2.50^**^	6.96^*^	3.98 ^NS^	− 7.15 ^NS^	− 12.87^*^	− 4.63^**^	− 9.25^**^
H18	L8 × T2	10.99^**^	− 2.75^**^	2.72 ^NS^	− 5.03 ^NS^	24.27^**^	17.28^**^	25.66^**^	17.08^**^
H19	L9 × T2	16.91^**^	3.66^**^	6.58	− 11.64^**^	− 30.79^**^	− 35.42^**^	− 30.91^**^	− 33.04^**^
H20	L10 × T2	9.73^**^	6.35^**^	9.78^**^	3.11 ^NS^	3.72 ^NS^	3.47 ^NS^	6.05^**^	4.25^**^
Minimum		− 0.15	− 1.28	− 0.70	− 2.09	− 3.95	− 0.80	− 3.48	− 2.88
Maximum		35.90	34.50	28.53	24.66	24.27	17.28	42.81	20.49

P.H. = plant height (cm); N.B.P. = number of branches plant^− 1^; D. F. = days to flowering (days); P.L. = pod length (cm); P.D. = pod diameter (cm), NS: non-significant, * and ** denotes significant at 0.05 and 0.01 levels of probability, respectively.

A.P.W.= average pod weight (g); N.P.P.= number of pods plant^− 1^; P.Y.P.= pod yield plant^− 1^ and T.Y.F.= total yield feddan^− 1^ (ton), NS: non-significant, * and ** denotes significant at 0.05 and 0.01 levels of probability, respectively.

Data in Table 3 represent the magnitudes of heterosis, versus mid parents and better parent, for average pod weight, number of pods plant^− 1^, pod yield plant^−1^and total yield feddan^− 1^. With respect to average pod weight, the F_1_ hybrids with significant positive heterosis were considered as desirable for pod weight. Crosse combinations L7×T1 (35.9%) followed by L2×T1 (19.37%), were found to be the highest for average pod weight over mid parents, whereas L4×T1 (34.50%) followed by L6×T2 (18.65) exhibited the highest magnituse of heterosis for pod weight over better parent. The average pod weight is a vital component in terms of total yield plant^− 1^^15^ and Chaudhary et al.,^5^. Additionally, the number of pods plant^− 1^, is one of the most major components of total yield which positive heterosis are preferred. Maximum heterosis magnitudes in descending order was recorded in the F_1_ hybrids L2 × T1 (28.53%) followed by L4×T1 (17.52%), L1×T1 (16.80%) over mid parent, while F_1_ hybrids L1×T1 (24.66%) followed by L2×T1 (15.60%), L5×T1 (8.19%) and L10 × T1 (8.09%) exhibited heterosis versus better parent for more number of pods plant^− 1^. Similar obtained results have been reported by^16–18^. With respect of pod yield per plant, seven F_1_ hybrids registered significant positive heterosis and four F_1_ hybrids registered significant negative heterosis versus mid parents. Meanwhile, significant positive heterosis was noticed in two F_1_ hybrids and four F_1_ hybrids exhibited significant negative heterosis versus better parent. Maximum pod yield was recorded for the F_1_ hybrids L8×T2 (24.27%) followed by L4×T2 (22.39%), L8×T1 (20.41%) and L6×T2 (17.39%) versus mid parents, whereas L8×T2 (17.28%) followed by L4×T2 (14.62%), L8×T1 (11.08%) and L5×T1 (7.08%) showed the maximum heterosis over better parent. High records of heterosis obtained in cross combinations unveiled considerable genetic variance between the parents and shows extend scope for commercial exploitation of heterosis in okra. Similar findings have been reported by Sood et al.,^45^ and Shinde et al.,^37^.

For total yield, fifteen F_1_ hybrids recorded significant positive heterosis and five F_1_ hybrids registered significant negative heterosis versus mid parents. Significant positive heterosis was noticed in twelve F_1_ hybrids and 8 F_1_ hybrids exhibited significant negative heterosis versus better parent. Heterosis values ranged from − 3.48 (H7) to 42.81% (H1) and − 2.88 (H6) to 20.49% (H8) both over mid parents and better parent, respectively. The maximum pod yield was recorded for the F_1_ hybrids L1×T1 (42.81%) followed by L8×T1 (29.86%), L8×T2 (25.66%) and L4×T2 (22.78%) versus mid parents and L8×T1 (20.49%) followed by L8×T2 (17.08%), L4×T2 (13.41%) and L10×T1(6.37%) over better parent. Similar results were found by ^11,13,38^.

It is concluded that the okra cultivars has potential to produce the heterotic hybrids and such cross combinations can be used for addition enhancements of okra traits. Some F_1_ hybrids, e.g., L1×T1, L8×T1, L8×T2, L4×T2, L4×T1 and L2×T2, showed heterotic actions for pod yield and other traits. This indicates that the hybrid can be exploited in okra breeding programs. Moreover, these cross-combinations could also be released as hybrids after further field test and their exploration in future breeding programs.

### General combining ability effects (*gi)*

General combining ability (GCA) effects, of ten lines and two testers, for the measured traits are displayed in Table 4. Data on the general combining ability effects of 12 parents together with average high performance increase the chance of obtaining suitable crosses. The obtained results cleared that many parents showed positive and significant magnitudes for the effect of general combining ability across different traits. L2, L3, L6, L7 and L10 showed positive and significant magnitudes for plant height, in the same table, the lines L1, L4, L5, L8 and L9 exhibited negative magnitudes and highly significant general combining ability effects. While L1, L3 and L6 displayed positive and significant magnitudes for number of branches plant^− 1^. With respect to, days to flowering, L1, L2 and L10 offered positive and significant values. The Lines L4 and L5 had negative significant (desirable) in the direction of earliness; the best general combiners for D.F. were L4 and L5. The tester (T2) had negative and significant GCA for days to flowering and it is a best general combiner to reduce the vegetative growth period. While, L5, L8 and L12 exhibited positive and significant magnitudes for pod length, Meanwhile, L8, L9 and L10 exhibited positive and significant values for pod diameter. As for pod weight, between the lines, L8 (1.30) recorded the highest positive and significant magnitudes *gca* effect and out of 10 lines, 5 exhibited negative gca effects and 5 exhibited positive *gca* effects, the *gca* effect varied from − 0.92 (L4) to 1.30(L8). For number of pods plant^− 1^, Out of 10 lines, the *gca* effect varied from − 10.11(L9) to 11.51 (L2). Out of 10 lines, 5 recorded negative significant general combining ability effects and 5 recorded positive significant general combining ability effects. On the other side, for total yield feddan^− 1^ L4, L5, L8 and L10 displayed positive and significant magnitudes, L4, L5, L8 and L10 it seems to be a promising general combiner. Other parents either exhibited negative or positive magnitudes that were not significant (referring to medium combining ability) or negative magnitudes of moral (referring to low combining ability) for the influence of general combining ability through the measured characters. It’s worth noting that crosses tend to show the behavior of parents they tend to, whether it’s to enhance or reduce the trait under this study. From a breeding perspective, such parents can be effectively utilized in developing superior hybrids or as donors in recurrent selection programs. These findings are in agreement with previous reports in okra, where parents with strong GCA effects were identified as key contributors to yield enhancement^14^. Similar conclusions have been reported in okra and related crops, where parents with high GCA were recommended for sustained genetic improvement.

**Table 4:** Estimation of general combining ability effects (*g*_*i*_) for nine measured traits in okra.

GCA effects	*P*.H. (cm)	*N*.B.*P*.	D.F. (days)	*P*.L. (cm)		*P*.D. (cm)	A.*P*.W. (g)	*N*.*P*.*P*.		*P*.Y.*P*.	T.Y.F. (ton)	
Lines												
L1	− 8.63^**^	0.67^**^	3.05^**^		0.003 ^NS^	− 0.003 ^NS^	0.30^**^	2.95^**^	− 0.97 ^NS^			− 0.03 ^NS^
L2	5.26^**^	− 0.67^**^	3.00^**^		− 0.30^**^	− 0.020 ^NS^	0.52^**^	11.51^**^	− 10.80^**^			− 0.17^**^
L3	18.75^**^	0.50^**^	1.72 ^NS^		− 1.13^**^	− 0.120^**^	− 0.72^**^	− 4.99^**^	− 50.30^**^			− 0.73^**^
L4	− 12.68^**^	0.16 ^NS^	− 4.72^**^		− 0.73^**^	− 0.136^**^	− 0.92^**^	10.12^**^	84.37^**^			1.12^**^
L5	− 13.24^**^	− 0.83^**^	− 6.61^**^		1.44^**^	− 0.136^**^	0.35^**^	− 0.72 ^NS^	21.70^**^			0.25^**^
L6	4.71^**^	0.50^**^	− 0.94 ^NS^		− 1.11^**^	− 0.171^**^	− 0.72^**^	− 10.05^**^	− 13.13^**^			− 0.22^**^
L7	12.15^**^	0.17 ^NS^	0.55 ^NS^		− 0.30^**^	− 0.036 ^NS^	− 0.52^**^	3.23^**^	− 43.14^**^			− 0.61^**^
L8	− 14.57^**^	− 0.50^**^	− 0.44 ^NS^		3.017^**^	0.263^**^	1.30^**^	− 3.44^**^	92.87^**^			1.52^**^
L9	− 8.35^**^	0.00	− 1.27 ^NS^		− 0.36^**^	0.113^**^	− 0.43^**^	− 10.11^**^	− 95.63^**^			− 1.36^**^
L10	16.60^**^	0.23 ^NS^	5.66^**^		− 0.53^**^	0.246^**^	0.84^**^	1.50 ^NS^	15.03^**^			0.23^**^
*SE ( gca for line)*	2.406	0.265	2.08		0.04	0.041	0.10	1.98	3.04			0.041
*SE (g*_*i*_ *-g*_*j*_ *) line*	3.403	0.374	2.94		0.12	0.058	0.14	2.80	4.29			0.058
**Testers**												
T1	− 7.94^**^	− 0.18 ^NS^	− 1.19^*^		− 0.12^**^	− 0.016 ^NS^	− 0.31^**^	− 1.62^**^	− 3.83^**^			− 0.03^**^
T2	7.94^**^	0.18 ^NS^	1.19^*^		0.12^**^	0.016 ^NS^	0.31^**^	1.62^**^	3.83^**^			0.03^**^
*SE ( gca for tester)*	1.076	0.118	0.93		0.04	0.018	0.04	0.88	1.36			0.018
*SE(g*_*i*_ *− g*_*j*_*) tester*	1.522	0.167	1.31		0.05	0.026	0.06	1.25	1.92			0.026

P.H. = plant height (cm); N.B.P. = number of branches plant^− 1^; D.F. = days to flowering (days); P.L. = pod length (cm); P.D. = pod diameter (cm); A.P.W. = average pod weight (g); N.P.P. = number of pods plant^− 1^; P.Y.P. = pod yield plant^− 1^ and T.Y.F. = total yield feddan^− 1^ (ton), NS: non-significant, *, **: significant at 0.05 and 0.01 levels of probability, respectively.

### Specific combining ability effects (*sij*)

Specific combining ability effects *(sij)* pertaining to the 20 F_1_ hybrids are shown in Table 5 for all traits .Specific combining ability can be either positive or negative and *sca* always indicating specific hybrid and never to particular parent by itself. Between the total 20 hybrids, F_1_ hybrids displaying high *sca* effect were selected from each trait, and the *gca* case of the lines of each crosses has been noticed as either high or low and from the obtained results it is shown that none of the cross combination exhibited consistently high *sca* effects for all the traits studied. According to El-Sherbeny et al.,^11^ and Singh and Arivazhagan^39^, hybrids with high means, desirable *SCA* values and involving at least one of the lines with high *GCA* would likely increase the concentration of desirable alleles to improve traits in okra. In terms of *SCA*, significant differences were recorded between the F_1_ hybrids for majority of the studied traits. The F_1_ Hybrid L5×T2 recorded the highest magnitude for the plant height (12.00), while F_1_ hybrid L7×T2 demonstrated the highest positive magnitude for *SCA* of the number of branches (1.50). With respect to, days to flowering hybrid L8×T1 and L8×T2 displayed the negative and significant specific combining ability effects (desirable) for earliness. Additionally, L10×T1 exhibited high positive magnitudes for pod length (50.00). Also, F_1_ hybrid L10×T2 offered the high specific combining ability of pod diameter (0.17). Regarding to yield traits, the F_1_ hybrid L6×T2 appeared the high *SCA* for average pod weight (1.01), while the F_1_ hybrid L1×T1 revealed high *SCA* for number of pods plant^− 1^. Also, F_1_ hybrid L9×T1 registered the highest *SCA* for pod yield plant^−1^and total yield feddan^− 1^ (29.33 and 0.38), respectively. Parents who have a positive *GCA* magnitude were expected to have the ability to combine to other parents to produce genotypes with high yield potential^5^. This highlights that high parental performance alone does not guarantee superior hybrid performance, and careful evaluation of hybrids is essential for commercial hybrid development.

**Table 5:** Estimation of specific combining ability effects (*s*_*ij*_) for nine measured traits in okra.

Cross no.	SCA effects due to crosses	*P*.H. (cm)	*N*.B.*P*.	D.F. (days)	*P*.L. (cm)	*P*.D. (cm)	A.*P*.W. (g)	*N*.*P*.*P*.	*P*.Y.*P*.	T.Y.F. (ton)
H1	L1 × T1	6.09^**^	0.67^**^	0.81 ^NS^	0.20^**^	0.12^**^	0.90^**^	5.89^**^	8.33^**^	0.10^**^
H2	L2 × T1	5.25^**^	0.00	3.50^*^	0.20^**^	0.13^**^	0.80^**^	4.78^**^	− 11.83^**^	− 0.18^**^
H3	L3 × T1	5.56^**^	− 0.17 ^NS^	− 1.02 ^NS^	0.41^**^	− 0.13^**^	− 0.44^**^	− 4.16^**^	− 23.33^**^	− 0.35^**^
H4	L4 × T1	− 0.88 ^NS^	0.50^*^	− 0.63 ^NS^	− 0.22^**^	0.02 ^NS^	− 0.41^**^	2.28 ^NS^	− 14.00^**^	− 0.19^**^
H5	L5 × T1	− 12.11^**^	0.50^*^	− 1.08 ^NS^	− 0.38^**^	0.08^*^	− 0.04 ^NS^	− 2.33 ^NS^	11.67^**^	0.11^**^
H6	L6 × T1	5.95^**^	0.50^*^	0.26 ^NS^	− 0.05 ^NS^	− 0.08^*^	− 1.01^**^	− 1.77 ^NS^	− 6.83^**^	− 0.12^**^
H7	L7 × T1	− 2.61 ^NS^	− 1.50^**^	− 2.02 ^NS^	0.12 ^NS^	0.05 ^NS^	0.03 ^NS^	0.17 ^NS^	6.50^**^	0.05
H8	L8 × T1	− 9.66^**^	− 0.83^**^	6.00^**^	0.12 ^NS^	0.05 ^NS^	0.11^*^	− 0.72 ^NS^	7.83^**^	0.12^**^
H9	L9 × T1	2.34 ^NS^	− 0.33 ^NS^	− 4.97^**^	0.22^**^	− 0.07*	− 0.96^**^	− 3.16^*^	29.33^**^	0.38^**^
H10	L10 × T1	0.17 ^NS^	0.67^**^	− 1.36 ^NS^	0.50^**^	− 0.17^**^	0.51^**^	− 0.99 ^NS^	8.00^**^	0.08^*^
H11	L1 × T2	− 6.06^**^	− 0.67^**^	− 0.81 ^NS^	− 0.20^**^	− 0.12^**^	− 0.90^**^	− 5.89^**^	− 8.33^**^	− 0.10^**^
H12	L2 × T2	− 5.17^**^	0.00	− 3.20^*^	− 0.20^**^	− 0.13^**^	− 0.80^**^	− 4.78^**^	11.83^**^	0.18^**^
H13	L3 × T2	− 5.56^**^	0.17 ^NS^	− 0.25 ^NS^	− 0.41^**^	0.13^**^	0.44^**^	4.16^**^	23.33^**^	0.35^**^
H14	L4 × T2	0.88 ^NS^	− 0.50^*^	0.63 ^NS^	0.22^**^	− 0.02 ^NS^	0.41^**^	− 2.28 ^NS^	14.00^**^	0.19^**^
H15	L5 × T2	12.00^**^	− 0.50^*^	1.08 ^NS^	0.38^**^	− 0.08^*^	0.04 ^NS^	2.33 ^NS^	− 11.67^**^	− 0.11^**^
H16	L6 × T2	− 5.95^**^	− 0.50^*^	− 0.26 ^NS^	0.05 ^NS^	0.08^*^	1.01^**^	1.77 ^NS^	6.83^**^	0.12^**^
H17	L7 × T2	2.61 ^NS^	1.50^**^	2.02 ^NS^	− 0.12 ^NS^	− 0.05 ^NS^	− 0.03 ^NS^	0.17 ^NS^	− 6.50^**^	− 0.05
H18	L8 × T2	9.66^**^	0.83^**^	− 5.53^**^	− 0.12 ^NS^	− 0.05 ^NS^	− 0.11^*^	0.72 ^NS^	− 7.83^**^	− 0.12^**^
H19	L9 × T2	− 2.34 ^NS^	0.33 ^NS^	4.97^**^	− 0.22^**^	0.07^*^	0.96^**^	3.16^*^	− 29.33^**^	− 0.38^**^
H20	L10 × T2	− 0.17 ^NS^	− 0.67^**^	1.36 ^NS^	− 0.50^**^	0.17^**^	− 0.51^**^	0.99 ^NS^	− 8.00^**^	− 0.08^*^
SE SCA		3.40	0.37	2.94	0.12	0.06	0.10	2.80	4.29	0.06
SE (S_ij_−S_jk_***)***		4.81	0.52	4.16	0.16	0.08	0.14	3.96	6.07	0.08

P.H. = plant height (cm); N.B.P.= number of branches plant^− 1^; D.F. = days to flowering (days); P.L. = pod length (cm); P.D. = pod diameter (cm); A.P.W= average pod weight (g); N.P.P.= number of pods plant^− 1^; P.Y.P. = pod yield plant^− 1^ and T.Y.F. = total yield feddan^− 1^(ton). NS: non-significant ; *, **: significant at 0.05 and 0.01 levels of probability, respectively.

### Genetic parameters

Genetic parameters for all the measured traits are presented in Table 6. The SCA variance magnitudes were higher than those of GCA variance for all measured traits, indicating that the non-additive genetic variance was major in controlling the inheritance of these traits. Researchers found similar results for different traits viz., El-Sherbeny et al.,^11^, Nanthakumar et al.,^26^ and Sidapara et al.,^38^. Furthermore, these results are also confirmed by the ratios of *σ*^*2*^*gca/ σ*^*2*^*sca* where traits having ratio of *σ*^*2*^*gca / σ*^*2*^*sca* < 1 indicate non-additive genetic variance, whereas the ratio > 1 depict the additive genetic variance. The ratio due to *σ*^*2*^*gca/ σ*^*2*^*sca* was lesser than zero for all measured traits under this study, which indicated the predominance of non-additive gene effect in the inheritance for these measured traits with low or moderate additive effects. So both hybridization and selection can be used for the improvement of these measured traits. Combining ability analysis revealed that non-additive variances were significant for pod yield plant^− 1^ and its contributing traits indicated their involvement in the expression of those traits. The magnitude of non-additive variance was higher for yield and its contributing traits indicated that non-additive gene effects were dominant and controlled these characters genetically.

The relative contributions of lines and testers and their interactions to the total variance indicated that the lines (female) contribution was significantly higher for all measured traits. The variance due to line x tester interaction was the most important contributor to the total variation in all major yield related traits when compared with either lines (female) or testers (male) variances. This indicated that upon crossing, enough genetic variation had been created for aforementioned characters in the collection under the experiment. The obtained data indicated that the values of the non-additive genetic variance (*σ*^*2*^*D*) were higher than the additive genetic variance (*σ*^*2*^*A*) for all the measured traits indicating the major role of non-additive gene effect in the inheritance of the studied traits. In addition, the estimates of thedegree of dominance (D.d.) showed values greater than unity for most of studied traits, indicating that the over-dominance was the most contributor of non-addititve gene actions which is favorable for heterosis breeding. However, the values of degree of dominance are nearly equal to one for pod yield/plant and total yield/plant, indicating a case of complete dominance. It can be assumed that the parents of these crosses carried more dominant alleles Moreover, the estimates of the heritability in broad sense were higher than those of narrow sense for all studied traits. The largest magnitude of heritability in broad sense (98.59%) was recorded for pod length, whereas the lowest magnitude (24.83%) was noticed for days to flowering. On the other hand, the estimates of heritability in narrow sense showed low to moderate estimations. The narrow sense heritability values varied from 0.49 to 47.37% for pod length and total yield feddan^− 1^, respectively. The presence of moderate narrow sense heritability indicate that majority of the traits are substantially governed by non-additive and environmental factors; therefore, selection efforts should be targeted to subsequent generations. Traits that exhibited high broad sense heritability reflect the high non-additive (dominance) genetic variation method, indicating the importance of hybridization method to improvement studied traits. These results ensure the predominance of non-additive genetic variance over additive genetic variance in the inheritance of the aforementioned traits. Similar results were confirmed by^11,30^.

**Table 6:** Estimations of genetic parameters for nine measured traits in okra.

Gen.Par. Traits	σ^2^ gca	σ^2^ sca	σ^2^ gca / σ^2^ sca	Contribution (%)			σ^2^A	σ^2^D	D.d.	h^2^_b.S_ %	h^2^_*n*.S_ %
				Line (%)	Tester (%)	Interaction (%)					
P.H. (cm)	12.30	73.48	0.17	59.97	24.90	15.13	24.60	73.48	1.73	73.84	18.52
N.B.P.	0.006	0.54	0.01	56.13	2.76	41.11	0.014	0.54	6.21	56.88	2.04
D.F. (days)	0.39	7.77	0.05	58.21	6.72	35.08	0.78	7.77	3.16	24.83	2.26
P.L. (cm)	0.006	2.78	0.002	97.86	0.75	1.39	0.014	2.78	14.09	98.59	0.49
P.D. (cm)	0.001	0.02	0.05	67.89	1.05	31.16	0.002	0.02	3.16	68.75	6.25
A.P.W. (g)	0.02	0.60	0.03	57.75	10.96	31.29	0.04	0.60	3.87	91.43	5.71
N.P.P.	2.92	14.67	0.20	79.68	4.04	16.28	5.84	14.67	1.58	46.62	13.28
P.Y.P.	200.08	461.74	0.43	92.92	0.45	6.63	400.16	461.74	1.07	93.97	43.63
T.Y.F. (ton)	0.04	0.09	0.44	94.00	0.14	5.86	0.08	0.09	1.06	94.74	47.37

P.H. = plant height (cm); N.B.P. = number of branches plant^− 1^; D.F. = days to flowering (days); P.L. = pod length (cm); P.D. = pod diameter (cm); A.P.W. = average pod weight (g); N.P.P. = number of pods plant^− 1^; P.Y.P. = pod yield plant^− 1^ and T.Y.F. = total yield feddan^− 1^ (ton).

## Conclusion

The present study revealed significant genetic variability among okra genotypes, indicating ample scope for genetic improvement. The results illustrated that non-additive gene action play a predominant role in the inheritance of yield and its associated traits, whereas additive gene action showed lesser effect in the expression of the studied traits. Several F_1_ hybrids, particularly L8xT2, L4xT2 and L8xT1, presented significant heterosis and superior yield performance. Promising parental lines viz., L4, L5, L8 and L10 showed high combining ability, making them suitable for hybrid development. The line × tester interaction was significant for all studied traits considered, refereeing that there were differences among hybrids. Thus, the promising hybrids which revealed desirable specific combining ability action and gave high evaluates of useful heterosis could be utilized for okra hybrids production. Overall, the findings indicate that exploiting heterosis through hybrid breeding is an effective strategy for improving okra yield, and the identified hybrids have potential for future breeding and commercialization after further breeding programs for the improvement of these traits in okra.

## Data Availability

The authors confirm that the data supporting the findings of this study are available within theArticle.
